# Characterization of Disease Progression in the Initial Stages of Retinopathy in Type 2 Diabetes: A 2-Year Longitudinal Study

**DOI:** 10.1167/iovs.61.3.20

**Published:** 2020-03-17

**Authors:** Inês P. Marques, Dalila Alves, Torcato Santos, Luís Mendes, Conceição Lobo, Ana Rita Santos, Mary Durbin, José Cunha-Vaz

**Affiliations:** 1 AIBILI—Association for Innovation and Biomedical Research on Light and Image, Coimbra, Portugal; 2 Department of Ophthalmology, University of Coimbra, Coimbra, Portugal; 3 Department of Orthoptics, Superior School of Health, Polytechnic of Porto, Porto, Portugal; 4 Advanced Development, Carl Zeiss Meditec, Inc., Dublin, California, United States

**Keywords:** diabetic retinopathy, optical coherence tomography, biomarker, progression

## Abstract

**Purpose:**

To characterize 2-year changes occurring in neurodegeneration, edema, and capillary dropout in nonproliferative diabetic retinopathy.

**Methods:**

Two-year prospective longitudinal observational cohort of eyes/patients with type 2 diabetes using spectral domain optical coherence tomography (SD-OCT) and optical coherence tomography angiography (OCTA). Eyes were examined three times with intervals of 1 year. Thickness of the full retina and layer-by-layer measurements were used to identify edema or neurodegeneration. OCTA vessel density maps of the retina were used to identify capillary dropout. Early Treatment Diabetic Retinopathy Study (ETDRS) classification was performed using the seven-field ETDRS protocol.

**Results:**

A total of 62 eyes from 62 patients with diabetes were followed for 2 years. After verification for image quality, a total of 44 eyes from 44 patients (30% women) aged 52 to 80 years were retained for data analysis. There were 18 eyes with ETDRS grades 10 to 20, 17 eyes with ETDRS grade 35, and 9 eyes with ETDRS grades 43 to 47. During the 2-year follow-up period, there was a progressive increase in capillary dropout, whereas edema and neurodegeneration remained stable. In multivariate analysis, considering a model adjusted for age, sex, hemoglobin A_1C_, visual acuity, and diabetes duration, vessel density remained significantly different between Diabetic Retinopathy Severity Scale groups (Wilks’ *λ* = 0.707; *P* = 0.015) showing association with disease progression.

**Conclusions:**

Capillary dropout increased in a period of 2 years in eyes with minimal, mild, and moderate diabetic retinopathy, whereas the presence of edema and neurodegeneration remained stable.

Optical coherence tomography (OCT) is a noninvasive imaging modality that allows detailed structural visualization of the retina. Optical coherence tomography angiography (OCTA) is a functional extension of OCT that detects motion or blood flow contrast. OCTA imaging has emerged as a noninvasive strategy to visualize the retinal and choroidal microvasculature without the use of an intravenous dye injection.^1^^,^^2^ It offers the possibility of quantification of features of interest. In particular, vessel density in the macula and the size of the foveal avascular zone (FAZ) are known to be affected by the presence of diabetic retinopathy (DR),^2^ allowing to discriminate between different eyes of different patients and correlate with clinically relevant measures such as stage of disease and visual acuity.^3^^,^^4^ Structural OCT has also shown in diabetic eyes the presence of neurodegeneration evidenced by thinning of the retinal nerve fiber layer (RNFL) or ganglion cell and inner plexiform layers (GCL-IPL) and the occurrence of edema evidenced by full retina thickening and localized thickening of the inner nuclear layer.^5^^,^^6^ OCT and OCTA offer, therefore, the possibility of quantifying the occurrence of retinal neurodegeneration, retinal edema, and capillary dropout (reduced vessel density) in the initial stages of DR. The fundus abnormalities seen in DR can be split into three categories: those findings resulting from increased apoptosis (neurodegeneration), those findings resulting from leaking microvasculature (retinal edema, hemorrhages), and those findings resulting from microvascular changes (decreased perfusion, ischemia).^7^^–^^9^

In a recent study, we have shown that the prevalence of different disease pathways varies between patients, even within the same severity group. Ischemia (capillary dropout) was the only disease pathway that showed correlation with retinopathy severity and metabolic control.^10^

For this analysis, we examined eyes of patients with type 2 diabetes and initial Non-Proliferative Diabetic Retinopathy (NPDR) three times with intervals of 1 year (baseline, 1-year, and 2-year visits). All eyes underwent seven-field fundus photography for ETDRS classification as well as spectral domain OCT (SD-OCT) and OCTA, looking for the occurrence of microvascular alterations, retinal neurodegeneration, or retinal edema. Presence of these alterations and their progression were analyzed during a 2-year period.

## Methods

In this prospective longitudinal observational cohort (ClinicalTrials.gov identifier: NCT03010397), participants with diabetes with initial stages of NPDR were analyzed from January 2016 to November 2018. The tenets of the Declaration of Helsinki were followed, and approval was obtained from the National Ethics Committee for Clinical Research (and Institutional Ethical Review Board) and written informed consent to participate in the study was obtained from all individuals after all procedures were explained. An age-matched population of individuals without diabetes or other retinal diseases was used as controls including 84 eyes (45 women and 39 men; mean [SD] age, 69.2 [4.5] years).

All participants underwent a full ophthalmologic examination, SD-OCT, and OCTA imaging. Seven-field color fundus photography was performed on diabetic participants for DR severity grading.

Exclusion criteria included any previous laser treatment or intravitreal injections, or presence of age-related macular degeneration, glaucoma, or vitreomacular disease and high ametropia (spherical equivalent greater than –6 and +2 diopters), or any other systemic disease that could affect the eye, with special attention for uncontrolled systemic hypertension (values outside normal range: systolic 70–210 mm Hg and diastolic 50–120 mm Hg) and history of ischemic heart disease. Eyes with central thinning and values identifying center-involved macular edema but without any evidence of cysts and no indication for treatment were included.

Age, duration of diabetes, hemoglobin A_1C_, and blood pressure level were collected for each participant from their patient records. The DR severity level was determined within the context of an experienced reading center and was based on the seven-field protocol using the ETDRS classification. Visual acuity was measured for each eye using the ETDRS protocol and Precision Vision charts at 4 m. A total of 62 eyes from 62 patients with diabetes were examined three times with intervals of 1 year (baseline, 1-year, and 2-year visits). The eyes were classified in three groups according to their Diabetic Retinopathy Severity Scale (DRSS): 10 to 20, 35, and 43 to 47. Of these eyes, only 44 eyes from 44 patients with diabetes passed the quality checks established for the three points of data collection performed at 1-year intervals. For data analysis, there were 18 eyes/patients with level 10 to 20, 17 eyes/patients for level 35, and 9 eyes/patients for level 43 to 47.

### Thinning and Thickening of the Retina Layers (Neurodegeneration and Edema)

The Macular Cube 512 × 128 acquisition protocol, consisting of 128 B-scans with 512 A-scans each, was used to assess the subjects’ central retinal thickness (CRT) and the average thickness value of the GCL-IPL collected from the standard CIRRUS examination reports.

CRT was used to identify eyes with (sub)clinical macular edema following the reference values established by the DRCR.net for CIRRUS SD-OCT.^11^^,^^12^ Clinical macular edema is defined as retinal thickness greater than or equal to 290 µm in women and greater than or equal to 305 µm in men, and subclinical macular edema is defined as retinal thickness between 260 µm and 290 µm in women and between 275 µm and 305 µm in men.

Retinal layer segmentation for layer thickness calculation was performed on the structural OCTA using the segmentation software implemented by AIBILI (Santos T, et al. *IOVS* 2015;43:ARVO E-Abstract 5953). Automated analysis results were reviewed by a masked grader.

RNFL and/or GCL-IPL thickness decreases were considered to identify neurodegeneration,^13^ whereas full retina thickness increases were considered to identify edema.^5^

### Capillary Dropout (Ischemia)

OCTA data were collected by the CIRRUS HD-OCT 5000 with AngioPlex OCT Angiography (Carl Zeiss Meditec, Dublin, CA, USA) device using the Angiography 3 × 3-mm^2^ acquisition protocol. To calculate the perfusion density and the vessel density, a thresholding algorithm was applied to the superior capillary plexus (SCP), deep capillary plexus (DCP), and full retina (FR) en face images to create a binary slab that assigns to each pixel a 1 (perfused) or 0 (background). From this slab, a skeletonized slab was created, representing vessels with a trace of 1 pixel in width. We define the perfusion density as the total area of perfused vasculature per unit area in a region of measurement, calculated by taking the mean of the binary slab within a desired region of interest. We define the vessel density as the total length of perfused vasculature per unit area in a region of measurement. A similar length-based metric has been used as a measurement of road density.^4^ We calculate the vessel density by taking the mean of the skeletonized slab within a desired region of interest and scaling the result by the distance between pixels (in this case, 245 pixels per 3 mm). The mean of the skeletonized slab is only a first-order estimate of the length of perfused vasculature. The Angiography 3 × 3-mm^2^ acquisition protocol consists of a set of 245 clusters of B-scans repeated four times, where each B-scan consists of 245 A-scans over a 3 × 3 × 2-mm^3^ volume in the central macula. The CIRRUS eye-tracking algorithm was used to reduce the effect of eye motion artifacts. For quality check, all OCTA acquisitions were reviewed by a masked grader. Only eyes that had OCTA examinations with signal strength greater or equal to 7, minimal motion artifacts, and no evidence of defocus or blur in the three examinations were included in this analysis. This is particularly relevant in a longitudinal study where data from different examinations are compared. From the total number of scans examined, 17% were excluded. As a result, from the total number of patients followed in the study, 29% were excluded from the data analysis because they did not meet the set of quality criteria in the three examinations leading to the final number of 44 patients that fulfilled the quality criteria in the three examinations.

Vessel density metrics for the entire 3 × 3-mm^2^ central macular area were computed for the SCP, DCP, and FR by the automated Carl Zeiss Density Exerciser software (version 10.0.0.12787). Area and circularity index of the FAZ for the SCP were also computed using the same software. The FAZ circularity index follows the 4πA/P^2^ ratio, with A being the area and P the perimeter. To account for potential projection artifacts, particularly when examining the DCP, we also used vessel density metrics of the FR.

Capillary dropout was therefore identified by decreased vessel density measured in the SCP, DCP, and FR.

### Statistical Analysis

Statistical analysis was performed with Stata 12.1 (Stata Corp. LP, College Station, TX, USA), and a *P* value ≤0.05 was considered statistically significant.

Reference values were taken considering the mean and standard deviation (SD) values of the healthy control population, taking the patient's sex into consideration.

Variables were summarized for each DRSS group, 10 to 20, 35, and 43 to 47, using the following statistics: mean, SD, confidence interval, and normalized mean (SD) difference from the control adjusted by sex.

To measure the association between two categorical variables, the *χ*^2^ test was used. Continuous variables were compared between groups using the ANOVA test. To explore correlations, the Pearson coefficient and the respective significance were computed. Mann-Whitney *U* tests were performed to assess the statistically significant difference between the measurements of healthy controls and diabetic patients, while to perform similar assessment within the same group of patients for the different visits, the Wilcoxon signed-rank test was used.

ANOVA and multivariate ANOVA were used to perform multivariate analysis to test associations between the study features and progression in disease severity adjusting for baseline characteristics.

## Results

**Figure 1.**
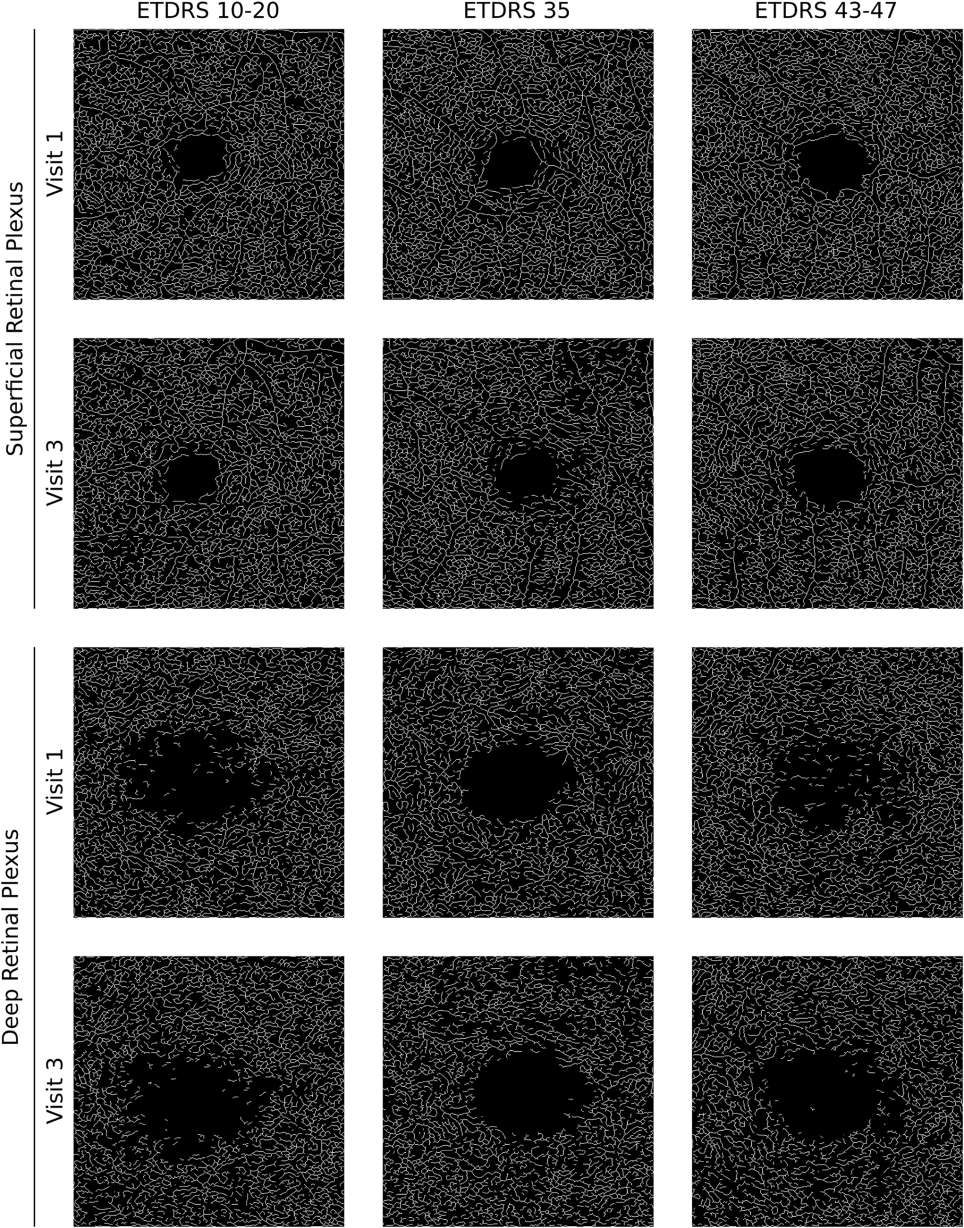
Examples of changes in vessel density of the superficial and deep retinal plexi during the 2-year follow-up period of three eyes, one from the DRSS 10 to 20 group, one from the DRSS 35 group, and another from the DRSS 43 to 47 group.

The demographic and baseline systemic and ocular characteristics of eyes/patients for each of the stages of the disease are presented in Table 1 and 2, respectively. No statistically significant systemic differences were found between eyes/patients within different ETDRS stages of the disease (Table 1). Only ocular changes such as vessel density in the SCP and FAZ circularity were significantly different between DRSS groups, reflecting an association between retinal capillary dropout and different severity stages of the disease (Table 2, Fig. 1).

**Table 1.** Baseline Characteristics for Patients’ Systemic Data Considering the Distinct DRSS Stages of the Disease

Characteristic	Controls(*n* = 84)	EDTRS 10–20(*n* = 18)	EDTRS 35(*n* = 17)	EDTRS 43–47(*n* = 9)	*P* Value(Three DRSS Groups)^*^
**Age, y**					
Mean (SD) [range]	69.2 (4.5)[59–84]	66.4 (6.6)[52–79]	64.9 (6.0)[54–78]	62.4 (8.5)[54–80]	0.368
Median (Q1–Q3)	68 (66–72)	66.5 (61.5–70.2)	65.6 (60.2–67.3)	59.3 (56.0–66.7)	
**Sex, No./total No. (%)**					
Female	45/84 (53.6)	8/18 (44.4)	2/17 (11.8)	3/9 (33.3)	0.102
Male	39/84 (46.4)	10/18 (55.6)	15/17 (88.2)	6/9 (66.7)	
**Diabetes duration, y**					
Mean (SD) [range]	–	17.9 (7.5)[6.8–34.8]	16.1 (5.9)[3.0–24.3]	16.1 (5.5)[6.8–24.1]	0.690
Median (Q1–Q3)	–	18.2 (12.3–24.4)	17.9 (11.4–19.8)	16.5 (14.7–20.1)	
**Hemoglobin A_1c_, %**					
Mean (SD) [range]	–	6.7 (0.9)[4.8–8.5]	6.9 (1.1)[4.2–8.6]	7.6 (1.2)[6.6–10.5]	0.070
Median (Q1–Q3)	–	6.7 (6.0–7.1)	6.9 (6.6–7.4)	7.4 (7.0–7.8)	
**BCVA, letters**					
Mean (SD) [range]	–	82.8 (5.2)[65–90]	84.1 (3.2)[75–90]	82.8 (4.4)[75–90]	0.614
Median (Q1–Q3)	–	85 (80–85)	85 (85–85)	85 (80–85)	

Q1–Q3, first to third quartiles; BCVA, best-corrected visual acuity.

* *P* value for the *χ*^2^ test and the ANOVA test for comparison between the three DRSS groups.

**Table 2.** Baseline Characteristics of Eyes/Patients Considering Distinct DRSS Stages of the Disease

Characteristic	Controls(*n* = 84)	EDTRS 10–20(*n* = 18)	EDTRS 35(*n* = 17)	EDTRS 43–47(*n* = 9)	*P* Value(Three DRSS Groups)^*^
**VD SCP, mm^−1^**					
Mean (SD) [range]	21.1 (0.7)[20.2–22.8]	20.5 (1.0)[18.3–21.9]	19.4 (1.6)[16.1–22.3]	19.5 (1.8)[15.8–21.9]	**0.047**
*P* value^†^		0.051	**<0.001**	**0.005**	
**VD DCP, mm^−1^**					
Mean (SD) [range]	16.1 (1.8)[12.2–19.8]	16.0 (1.5)[13.8–19.5]	15.5 (2.1)[10.5–19.8]	14.8 (2.9)[9.1–17.9]	0.386
*P* value^†^		0.632	0.257	0.005	
**VD FR, mm^−1^**					
Mean (SD) [range]	22.4 (0.6)[21.2–24.2]	22.2 (0.8)[20.4–23.3]	21.4 (1.5)[18.2–24.0]	21.1 (1.6)[17.6–23.3]	0.072
*P* value^†^		0.510	**<0.001**	**0.006**	
**FAZ area, mm^2^**					
Mean (SD) [range]	0.24 (0.11)[0.04–0.54]	0.27 (0.12)[0.10–0.55]	0.23 (0.12)[0.07–0.43]	0.23 (0.08)[0.14–0.37]	0.440
*P* value^†^		0.309	0.717	0.907	
**FAZ circularity index**					
Mean (SD) [range]	0.65 (0.07)[0.39–0.87]	0.68 (0.06)[0.58–0.79]	0.62 (0.09)[0.39–0.75]	0.60 (0.10)[0.37–0.70]	**0.019**
*P* value^†^		0.104	0.260	0.887	
**RNFL thickness, µm**					
Mean (SD) [range]	7.0 (3.4)[0.7–13.6]	6.3 (2.9)[2.5–13.7]	7.1 (3.4)[2.3–15.1]	6.3 (1.8)[4.6–9.9]	0.691
*P* value^†^		0.449	0.889	0.556	
**GCL-IPL thickness, µm**					
Mean (SD) [range]	82.7 (5.5)[71–94]	82.0 (6.6)[72–92]	76.2 (7.4)[58–90]	81.3 (8.4)[69–97]	0.056
*P* value^†^		0.878	**0.001**	0.484	
**Full retinal thickness, µm**					
Mean (SD) [range]	260.6 (18.3)[218–299]	261.6 (25.2)[212–300]	270.4 (30.1)[225–320]	267.0 (27.0)[223–304]	0.565
*P* value^†^		0.669	0.365	0.919	

VD, Vessel Density. Values in bold indicate statistical significance.

* *P* value for the Mann-Whitney test for comparison between the control and DRSS groups.

† *P* value for the ANOVA test for comparison between the three DRSS groups.

In each DRSS group, values for capillary dropout (reduced vessel density), edema, and neurodegeneration covered a wide range, identifying different levels of damage in different eyes as shown by the maximum and minimum values found for each of the variables (Table 2).

The presence of capillary dropout, evidenced by reduced vessel density in SCP, DCP, and FR, showed statistically significant differences at 1- and 2-year intervals (Table 3). Moreover, in the SCP, capillary dropout was found to be significant in all ETDRS levels examined after the 2-year period.

**Table 3.** Progression in Capillary Dropout, Neurodegeneration, and Edema After 1- and 2-Year Intervals in Different DRSS Groups

		Visit 1	Visit 2	Visit 3	*P* Value	*P* Value
Characteristic		Mean (SD)	Mean (SD)	Mean (SD)	V2–V1^*^	V3–V1^*^
**ETDRS 10–20 (*n* = 18)**						
Vessel density, mm^−1^	SCP	20.5 (1.0)	19.4 (1.3)	18.8 (1.5)	**0.002**	**<0.001**
	DCP	16.0 (1.5)	14.7 (2.1)	13.9 (1.9)	**0.012**	**0.001**
	FR	22.2 (0.8)	21.3 (1.1)	20.7 (1.2)	**0.003**	**0.001**
FAZ (circularity index)		0.7 (0.1)	0.7 (0.1)	0.6 (0.1)	0.102	**0.031**
RNFL (thinning, µm)		6.3 (2.9)	6.0 (2.8)	6.0 (3.0)	0.948	0.647
GCL-IPL (thinning, µm)		82.0 (6.6)	81.4 (6.1)	81.7 (6.6)	**0.024**	0.209
Full retina (thickening, µm)		261.6 (25.2)	261.7 (26.5)	261.8 (26.2)	0.742	0.660
**ETDRS 35 (*n* = 17)**						
Vessel density, mm^−1^	SCP	19.4 (1.6)	18.6 (2.1)	17.9 (1.7)	0.062	**0.001**
	DCP	15.5 (2.1)	14.8 (2.5)	13.7 (2.3)	**0.028**	**<0.001**
	FR	21.4 (1.5)	20.5 (2.0)	20.0 (1.6)	**0.015**	**0.001**
FAZ (circularity index)		0.6 (0.1)	0.6 (0.1)	0.6 (0.1)	0.089	0.754
RNFL (thinning, µm)		7.1 (3.4)	7.1 (3.9)	6.0 (3.0)	0.981	0.136
GCL-IPL (thinning, µm)		76.2 (7.4)	76.5 (6.7)	76.5 (7.2)	0.391	0.337
Full retina (thickening, µm)		270.4 (30.1)	270.5 (29.2)	271.1 (29.9)	0.226	0.115
**ETDRS 43–47 (*n* = 9)**						
Vessel density, mm^−1^	SCP	19.5 (1.8)	18.9 (1.6)	17.9 (2.4)	0.110	**0.015**
	DCP	14.8 (2.9)	14.1 (2.9)	13.3 (2.6)	0.173	0.066
	FR	21.1 (1.6)	20.7 (1.6)	19.5 (2.1)	0.110	**0.015**
FAZ (circularity index)		0.6 (0.1)	0.6 (0.1)	0.5 (0.1)	0.953	0.069
RNFL (thinning, µm)		6.3 (1.8)	6.9 (2.4)	6.7 (4.5)	0.767	0.767
GCL-IPL (thinning, µm)		81.3 (8.4)	81.7 (8.2)	81.7 (8.3)	0.257	1.0
Full retina (thickening, µm)		260.7 (27.0)	262.7 (29.0)	263.3 (28.5)	0.372	0.169
**All patients**						
Vessel density, mm^−1^	SCP	19.9 (1.5)	19.0 (1.7)	18.3 (1.8)	**<0.001**	**<0.001**
	DCP	15.6 (2.1)	14.6 (2.4)	13.7 (2.2)	**<0.001**	**<0.001**
	FR	21.7 (1.3)	20.9 (1.6)	20.2 (1.6)	**<0.001**	**<0.001**
FAZ (circularity index)		0.6 (0.1)	0.6 (0.1)	0.6 (0.1)	**0.034**	**0.025**
RNFL (thinning, µm)		6.6 (2.9)	6.6 (3.2)	6.1 (3.3)	0.944	0.294
GCL-IPL (thinning, µm)		79.6 (7.6)	79.6 (7.1)	79.7 (7.5)	0.628	0.884
Full retina (thickening, µm)		264.8 (27.3)	265.3 (27.7)	265.7 (27.8)	0.126	0.065

^*^ *P* value calculated by Wilcoxon signed-rank test. Values in bold indicate statistical significance.

Vessel density in the SCP, DCP, and FR showed strong positive correlations (SCP vs DCP: *r* = 0.77; *P* < 0.001; SCP vs FR: *r* = 0.96; *P* < 0.001; DCP vs FR: *r* = 0.84; *P* < 0.001). Also, vessel density decreased progressively, during the 2-year follow-up period, when analyzing the SCP, DCP, and FR. Still, the decreases were more apparent in SCP, particularly in the initial stages of DR (Table 3, Supplementary Table S1).

Progression in capillary dropout occurred after 1- and 2-year intervals in DRSS groups 10 to 20 and 35 but in groups 43 to 47, progression in capillary dropout, represented by decreased vessel density, was only identified after 2 years of follow-up (Table 3). In each DRSS group examined, there was a subgroup of eyes/patients that showed more marked decreases in vessel density (Table 4). We established a 10% decrease as relevant, as it equals a decrease of 3 standard deviations in the value of the control reference (healthy eyes; vessel density = 21.1 ± 0.7).

**Table 4.** Eyes Showing Decreases in Vessel Density of More Than 10% in the 2-Year Period

Visit	Vessel Density	EDTRS 10–20, % (No./Total No.)	EDTRS 35, % (No./Total No.)	EDTRS 43–47, % (No./Total No.)
V3–V1	SCP	27.8 (5/18)	41.2 (7/17)	22.2 (2/9)
	DCP	50.0 (9/18)	58.8 (10/17)	55.6 (5/9)
	FR	22.2 (4/18)	41.2 (7/17)	22.2 (2/9)

In multivariate analysis, considering a model adjusted for age, sex, hemoglobin A_1C_, visual acuity, and diabetes duration, vessel density remained significantly different between DRSS groups (Wilks’ *λ* = 0.707; *P* = 0.015) showing an association with disease progression.

**Figure 2.**
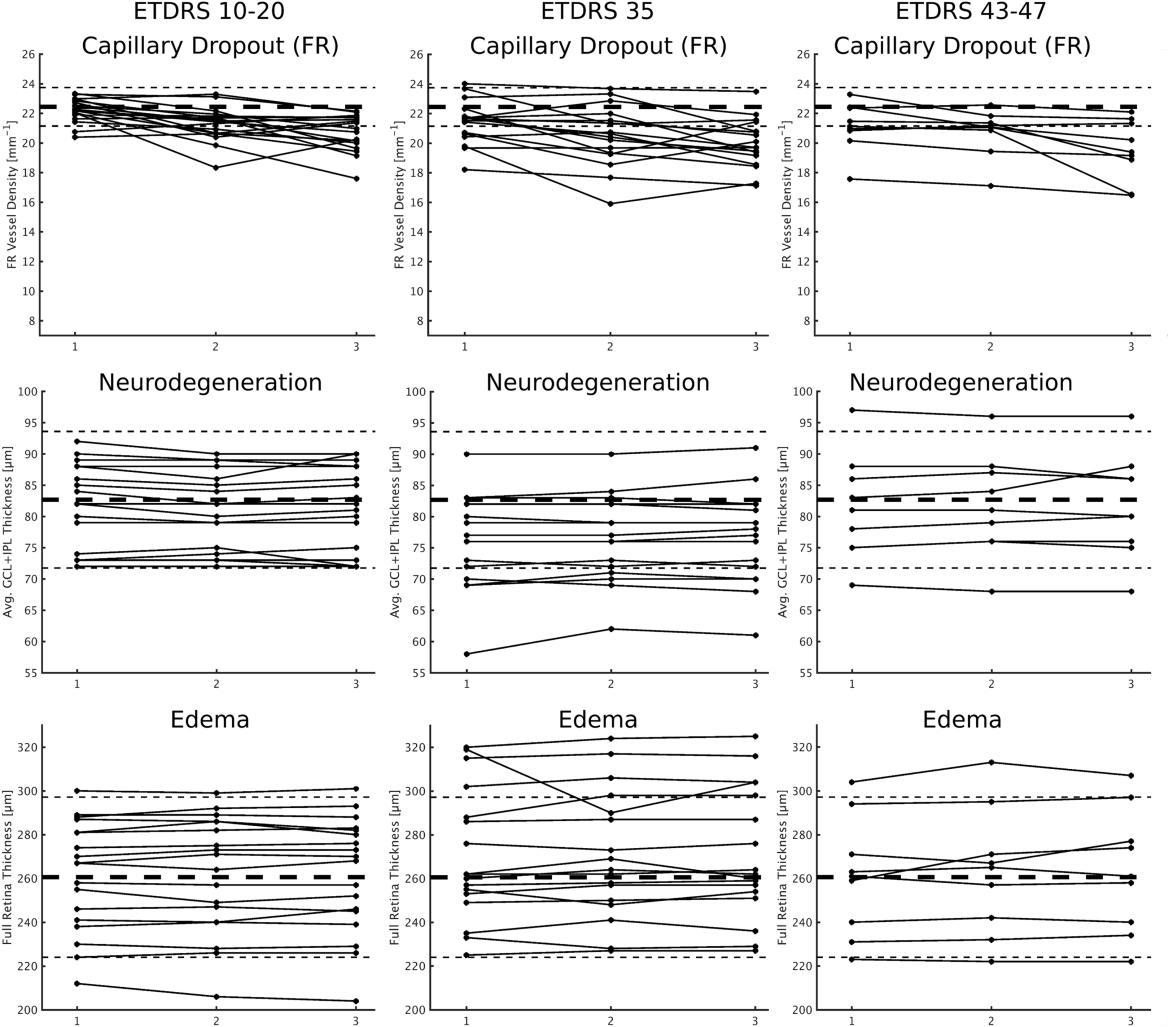
Individual changes over time, by ETDRS level at baseline, of vessel density metrics (SCP, DCP, and FR) for capillary dropout. *Horizontal black dashed lines* represent the average of the healthy control population (*thicker*) and the 2 standard deviations from the mean (*thinner*).

The differences between visits in full retina and layer thickness showed that neurodegeneration and edema remained generally stable during the 2-year period (RNFL: *P* = 0.504; GCL + IPL: *P* = 0.777; CRT: *P* = 0.480) (Fig. 2). Neurodegeneration was better identified in the GCL + IPL than RNFL.

When analyzing ETDRS level changes in the 2-year period of follow-up, there was only one eye in groups 10 to 20 (2%) with a two-step worsening change (10 to 35) (Supplementary Table S1). All other ETDRS changes were one-step changes, 10 eyes with worsening changes and 5 eyes with improvement changes. In the DRSS 10 to 20 group, there was one-step worsening in eight eyes and one-step improvement in one eye. In the DRSS 35 group, there was one-step worsening in one eye and one-step improvement in one eye. In the DRSS 43 to 47 group, there was one-step worsening in one eye and one-step improvement in three eyes. Although there was higher percentage of decrease in vessel density in the eyes that showed DRSS worsening (5.2% ± 4.8%) compared with DRSS improvement (3.7% ± 5.1%), a clear correlation between clinical progression (change in DRSS grade) and change in vessel density could not be identified due to the small number of cases with worsening and improvement in each grade.

When using the 10% vessel density decrease as a threshold, progression in the severity of diabetic retinopathy was identified in 9 of the 26 eyes/patients examined (35%), showing that vessel density has more than four times detection capacity than the ETDRS one-step worsening.

## Discussion

The results here reported confirm that eyes in the initial stages of retinopathy in patients with type 2 diabetes show evidence of neurodegenerative changes, edema, and capillary dropout and that these changes are present in different degrees in different patients. Furthermore, the metrics of these changes show a wide range of values. Definite neurodegeneration, edema, or capillary dropout can occur very early in the disease process but are not present in every patient and, when present, not at the same time or with the same degree.^10^

In this study, we have followed, for a period of 2 years, eyes categorized as minimal, mild, and moderate retinopathy using seven-field ETDRS grading. Of the three different disease pathways, only ischemia (capillary dropout), identified by metrics of vessel density using OCTA, showed significant progression. This progression in capillary dropout appears to be driven by a subgroup of eyes/patients that showed changes in vessel density of 10% or more from the baseline. These findings reinforce previous observations suggesting that increased capillary dropout (ischemia) is associated with increased severity of the retinopathy.^4^

The decrease in vessel density occurs in both retinal capillary plexi, superficial and deep, but the changes occurring in the superficial retinal capillary plexus are more reliably detected, evidenced by the lower standard deviation of the measurements performed in the SCP. To account for projections artifacts that may mask the detection of vessel density changes in the DCP, we also measured vessel density in the FR, detecting SCP and DCP changes simultaneously. The results obtained confirm earlier detection of capillary dropout in the SCP.^4^ This finding confirms that one of the earliest changes associated with diabetic retinopathy is a decrease in retinal blood flow.^14^^,^^15^

It is of major relevance that metrics of neurodegeneration and edema do not show progression over the 2-year period of follow-up. These disease pathways do not appear to be associated with increased severity of the retinopathy, even in eyes that showed one-step progression in ETDRS level. To identify retinal neurodegeneration, ganglion cell and inner plexiform layer measurements were more informative in this study than retinal nerve fiber layer measurements.

This study shows that only capillary dropout (ischemia) appears to be associated with increased severity of the retinopathy and 2-year progression of the retinal disease. It is noteworthy that, in a 2-year period of follow-up, minimal, mild, and moderate retinopathy show decreases in vascular density. During this 2-year follow-up study, there were mainly one-step changes in ETDRS grading, which are not generally accepted as clinically meaningful. Therefore, when using ETDRS level changes, a 2-year period of follow-up is clearly too short, as shown in this study. Metrics of vessel density identifying capillary dropout may offer a viable alternative to identify a specific phenotype associated with increased risk of progression.^16^^,^^17^

A strict quality check on OCTA vessel density measurements is particularly necessary when comparing different examinations performed in the same patient in longitudinal studies. In this study, with examinations performed by experienced technicians, data from 17% of the examinations performed could not be included in the data analysis because they did not pass the final quality check. Future longitudinal studies using OCTA for measurement of vessel density need to set strict standards for image quality that need to be checked at the end of each examination.

We have identified the presence of capillary dropout in the initial stages of diabetic retinal disease and that capillary dropout is associated with retinopathy severity and 2-year progression. Initially, the DCP, unlike the SCP, did not show capillary dropout, probably due to limitations in the measurement methodology. In this study, the decrease in retinal blood flow is better identified in the SCP and well identified also when measuring full retina vessel density metrics. The methods used for calculating DCP capillary density metrics may need to be refined, as suggested by Rosen et al.^18^ and Zhu et al.^19^

Limitations of this study are the number of eyes included in the study and the use of automated layer segmentation analyses for measurements of retinal thinning (neurodegeneration) and increased retinal thickness (edema). These procedures, however, were performed in retinas in the initial stages of retinal disease that remained structurally preserved with no evidence of cystoid changes and were reviewed by a masked grader.

In conclusion, eyes with minimal, mild, or moderate DR followed with repeated visits, for a period of 2 years, show evidence of neurodegeneration, edema, and ischemia, distributed over a wide range of values in different eyes/patients. Only capillary dropout (ischemia) showed evidence of progression over the 2-year period. This progression was particularly clear in a subgroup of eyes/patients showing higher rates of reduction in vessel density. This group of patients should receive particular attention, needing earlier diagnosis and closer follow-up. Metrics of vessel density obtained with OCTA in a noninvasive manner, allowing repeated examinations and close follow-up, will most likely be an informative biomarker of diabetic retinopathy progression and thus create the adequate environment for a larger role of precision medicine in the management of DR.

## Supplementary Material

Supplement 1
