# Supplementary material for: Characterization of Disease Progression in the Initial Stages of Retinopathy in Type 2 Diabetes: A 2-Year Longitudinal Study
Source: Invest Ophthalmol Vis Sci. 2020 Mar 17;61(3):20. doi: 10.1167/iovs.61.3.20 (PMC7401457; doi:10.1167/iovs.61.3.20)
Supplement: Supplement 1 [file iovs-61-3-20_s001.pdf]

ID	Gender	Age [yrs]	Diab. Dur. [yrs]	Visit	HbA1c [%]	Blood Pressure [mmHg]		BVCA	ETDRS	VD SRP		VD DRP		VD FR		FAZ Area		FAZ Circ		RNFL Thickness		GCL-IPL Thickness		Full Retina Thickness	
						S.	D.			[mm^{-1}]	%	[mm^{-1}]	%	[mm^{-1}]	%	[mm^2]	%	[au]	%	[µm]	%	[µm]	%	[µm]	%
				V1	7.3			65	20	20.1	--	14.6	--	22.1	--	0.2	--	0.6	--	6.8	--	73	--	267	--
1	Male	70	27	V2	7.3	147	75	66	20	19.6 *	-2.3	16.4	12.6	21.6	-2.4	0.2	15.6	0.7	22.1	7.8	13.4	74	1.4	271	1.5
				V3	7.4			85	35C	18.6 *	-7.2	16.9	15.7	20.1 *	-9.1	0.2	-2.4	0.6	0.1	7.1	3.2	75	2.7	270	1.1
				V1	5.8			80	10	21.1	--	15.4	--	22.3	--	0.4	--	0.7	--	2.7	--	82	--	241	--
2	Female	76	7	V2	6.4	142	71	80	10	19.9	-5.8	14.3	-7.3	21.4	-3.7	0.4	2.1	0.7	-2.5	1.9	-27.6	82	0.0	240	-0.4
				V3	6.4			85	35C	19.1 *	-9.3	12.6	-18.2	20.7 *	-6.9	0.4	-4.7	0.7	0.3	3.2	22.3	82	0.0	239	-0.8
				V1	6.9			85	10	21.6	--	17.4	--	23.3	--	0.2	--	0.7	--	13.7	--	84	--	274	--
3	Female	63	25	V2	6.9	147	81	85	10	19.6 *	-9.1	15.1	-13.3	22.2	-5.0	0.2	1.2	0.8	0.9	5.6	-59.3	82	-2.4	275	0.4
				V3	7.6			85	10	17.5 *	-18.6	12.5 *	-28.5	19.6 *	-15.9	0.3	9.0	0.7	-7.6	5.9	-56.8	83	-1.2	276	0.7
				V1	6.2			85	20	21.5	--	19.5	--	23.0	--	0.2	--	0.6	--	11.4	--	92	--	287	--
4	Female	52	19	V2	6.2	140	80	85	35C	21.6	0.4	19.6	0.5	23.3	1.4	0.2	2.8	0.7	6.2	12.5	10.1	90	-2.2	286	-0.3
				V3	6.2			85	35C	20.9	-2.8	18.7	-4.4	22.1	-3.9	0.2	1.9	0.6	-1.1	6.7	-41.0	90	-2.2	282	-1.7
				V1	6.5			85	10	21.0	--	16.2	--	22.5	--	0.3	--	0.7	--	6.7	--	88	--	258	--
5	Male	63	12	V2	6.5	136	67	90	10	20.5	-2.3	14.8	-8.6	21.9	-2.5	0.2	-6.4	0.8	12.2	5.8	-13.4	88	0.0	257	-0.4
				V3	6.5			85	10	19.1 *	-8.9	13.6	-16.2	21.0 *	-6.9	0.3	17.7	0.5	-22.4	6.2	-8.3	88	0.0	257	-0.4
				V1	6			85	10	21.8	--	16.3	--	23.3	--	0.3	--	0.6	--	7.1	--	74	--	281	--
6	Male	69	24	V2	6.7	125	67	85	10	21.5	-1.1	17.1	4.7	23.1	-0.7	0.3	6.0	0.6	-5.7	8.5	20.0	75	1.4	286	1.8
				V3	6.7			80	20	20.8	-4.3	15.2	-6.9	22.1	-4.9	0.2	-3.7	0.7	22.3	7.2	1.2	72	-2.7	280	-0.4
				V1	7			85	20	19.6 *	--	17.1	--	22.0	--	0.2	--	0.7	--	5.3	--	79	--	255	--
7	Female	62	17	V2	7	150	89	85	35D	17.3 *	-11.6	13.9	-18.6	19.8 *	-10.0	0.2	2.6	0.6	-5.9	7.2	37.2	79	0.0	249	-2.4
				V3	7.4			85	20	15.1 *	-22.6	12.1 *	-29.1	17.6 *	-20.2	0.1	-42.8	0.5	-26.5	7.3	37.8	79	0.0	252	-1.2
				V1	7.1			85	20	20.7	--	16.1	--	22.2	--	0.3	--	0.7	--	3.8	--	86	--	230	--
8	Male	61	15	V2	7.8	140	75	85	20	18.6 *	-10.1	12.6	-21.6	20.6 *	-6.9	0.4	6.4	0.6	-14.3	3.4	-10.4	85	-1.2	228	-0.9
				V3	7.8			88	20	19.7 *	-4.8	14.7	-8.5	21.5	-3.2	0.3	0.0	0.7	3.2	4.7	24.9	86	0.0	229	-0.4
				V1	6.5			85	20	20.2	--	13.8	--	21.6	--	0.5	--	0.7	--	6.2	--	85	--	224	--
9	Female	66	7	V2	6.1	142	72	85	20	20.4	0.7	13.9	1.1	21.7	0.4	0.4	-1.5	0.7	-1.0	5.0	-20.0	84	-1.2	226	0.9
				V3	6.4			85	35C	17.2 *	-14.8	12.1 *	-12.2	19.1 *	-11.5	0.4	-15.3	0.5	-35.1	0.7	-88.8	85	0.0	226	0.9
				V1	6			80	20	18.3 *	--	15.3	--	20.4 *	--	0.2	--	0.7	--	7.9	--	73	--	289	--
10	Male	73	9	V2	6.6	144	74	80	20	18.5 *	1.6	13.3	-13.5	20.6 *	1.2	0.2	3.5	0.6	-9.7	5.1	-35.0	73	0.0	289	0.0
				V3	6.7			80	10	17.2 *	-5.5	13.9	-9.4	19.4 *	-4.7	0.2	4.6	0.6	-9.2	7.3	-7.8	73	0.0	288	-0.3
				V1	8.5			80	20	20.5	--	15.7	--	22.0	--	0.6	--	0.7	--	4.2	--	80	--	212	--
11	Female	60	35	V2	8.4	145	77	80	20	16.4 *	-19.8	11.1 *	-28.9	18.3 *	-16.8	0.5	-2.5	0.6	-6.7	1.5	-64.4	79	-1.3	206	-2.8
				V3	8.8			85	20	18.3 *	-10.6	11.3 *	-28.1	20.2 *	-8.1	0.5	-16.1	0.4	-35.0	2.4	-42.1	80	0.0	204	-3.8

ID	Gender	Age [yrs]	Diab. Dur. [yrs]	Visit	HbA1c [%]	Blood Pressure [mmHg] S.	D.	BVCA	ETDRS	VD SRP [mm^{-1}]	%	VD DRP [mm^{-1}]	%	VD FR [mm^{-1}]	%	FAZ Area [mm^2]	%	FAZ Circ [au]	%	RNFL Thickness [µm]	%	GCL-IPL Thickness [µm]	%	Full Retina Thickness [µm]	%
				V1	4.8			90	20	21.4	--	17.1	--	22.8	--	0.2	--	0.7	--	8.3	--	86	--	300	--
12	Male	65	17	V2	4.8	114	74	85	35C	20.1	-6.0	16.0	-6.3	21.7	-5.0	0.2	4.9	0.7	-2.4	9.0	8.6	85	-1.2	299	-0.3
				V3	5.3			73	35C	19.8	-7.5	15.9	-7.0	21.8	-4.7	0.2	0.7	0.8	3.9	14.8	79.0	86	0.0	301	0.3
				V1	7.8			80	10	18.9 *	--	13.9	--	21.4	--	0.2	--	0.7	--	6.0	--	73	--	270	--
13	Male	67	20	V2	7.8	145	80	85	35C	18.1 *	-4.1	13.3	-4.5	20.9 *	-2.3	0.2	19.9	0.6	-18.9	4.2	-30.1	73	0.0	273	1.1
				V3	7.8			85	10	17.2 *	-9.1	12.9	-7.5	20.0 *	-6.6	0.2	29.3	0.7	-6.0	7.1	17.8	72	-1.4	273	1.1
				V1	7.9			85	20	21.9	--	15.3	--	22.7	--	0.3	--	0.7	--	3.8	--	90	--	246	--
14	Male	71	10	V2	8.7	143	71	85	20	19.4 *	-11.5	11.0 *	-28.2	20.4 *	-10.3	0.3	16.7	0.5	-21.3	4.2	9.7	89	-1.1	247	0.4
				V3	8.6			85	20	20.3	-7.6	11.9 *	-21.7	21.4	-6.0	0.3	7.6	0.5	-21.0	4.7	23.8	88	-2.2	245	-0.4
				V1	5.9			85	20	20.3	--	15.5	--	22.5	--	0.1	--	0.6	--	7.0	--	72	--	288	--
15	Female	79	19	V2	5.9	136	62	85	20	19.4 *	-4.2	14.3	-7.8	21.5	-4.4	0.1	21.8	0.5	-20.3	9.3	32.1	72	0.0	292	1.4
				V3	5.9			85	35C	19.4 *	-4.2	15.7	1.2	21.6	-4.0	0.1	42.7	0.5	-8.1	7.9	12.0	72	0.0	293	1.7
				V1	6.8			80	20	21.4	--	18.7	--	23.0	--	0.2	--	0.8	--	6.5	--	89	--	281	--
16	Male	69	19	V2	6.8	146	65	83	20	18.9 *	-11.8	16.1	-14.0	20.7 *	-10.1	0.2	8.8	0.7	-13.2	7.1	9.0	89	0.0	282	0.4
				V3	6.9			82	20	18.4 *	-14.0	13.7	-26.5	20.2 *	-12.3	0.2	10.3	0.5	-31.4	7.6	16.2	89	0.0	283	0.7
				V1	6.9			85	20	19.6 *	--	15.5	--	20.8 *	--	0.5	--	0.6	--	2.5	--	88	--	238	--
17	Female	70	14	V2	8.5	136	67	70	20	20.0	2.3	14.6	-6.0	21.3	2.6	0.5	0.3	0.7	10.9	5.6	123.2	86	-2.3	240	0.8
				V3	7.2			80	35C	20.5	4.5	13.6	-11.9	21.9	5.3	0.5	0.6	0.8	21.9	2.2	-12.3	90	2.3	246	3.4
				V1	6			85	10	20.1	--	15.1	--	21.9	--	0.3	--	0.7	--	3.4	--	82	--	267	--
18	Male	57	25	V2	6.2	121	56	80	10	19.9	-0.7	16.9	11.9	21.8	-0.5	0.2	-1.3	0.7	-3.7	4.6	35.0	80	-2.4	264	-1.1
				V3	6			80	20	20.0	-0.7	13.8	-8.6	21.7	-0.9	0.3	7.8	0.7	-8.8	5.0	46.2	81	-1.2	268	0.4

ID	Gender	Age [yrs]	Diab. Dur. [yrs]	Visit	HbA1c [%]	Blood Pressure [mmHg] S.	Blood Pressure [mmHg] D.	BVCA	ETDRS	VD SRP [mm^{-1}]	VD SRP %	VD DRP [mm^{-1}]	VD DRP %	VD FR [mm^{-1}]	VD FR %	FAZ Area [mm^{2}]	FAZ Area %	FAZ Circ [au]	FAZ Circ %	RNFL Thickness [µm]	RNFL Thickness %	GCL-IPL Thickness [µm]	GCL-IPL Thickness %	Full Retina Thickness [µm]	Full Retina Thickness %
19	Male	67	3	V1	4.2	127	75	80	35C	20.0	--	16.1	--	22.3	--	0.1	--	0.6	--	10.7	--	73	--	302	--
				V2	5.4			80	43A	17.1 *	-14.4	13.9	-14.0	19.3 *	-13.4	0.1	-56.6	0.5	-4.7	14.1	31.0	72	-1.4	306	1.3
				V3	6.6			85	35E	16.5 *	-17.4	13.8	-14.1	18.4 *	-17.4	E	E	E	E	7.6	-29.5	73	0.0	304	0.7
20	Male	71	21	V1	7.3	132	90	85	35C	18.1 *	--	13.4	--	19.8 *	--	0.4	--	0.7	--	4.8	--	70	--	225	--
				V2	7.2			76	35C	14.4 *	-20.1	10.8 *	-19.8	15.9 *	-19.6	0.4	2.4	0.6	-4.2	1.5	-68.5	69	-1.4	227	0.9
				V3	7.8			76	35C	15.5 *	-14.4	10.5 *	-21.7	17.3 *	-12.7	0.0	-99.8	0.9	39.2	2.0	-59.3	68	-2.9	227	0.9
21	Male	60	19	V1	8.5	128	78	85	35C	18.1 *	--	15.4	--	20.6 *	--	0.4	--	0.5	--	3.5	--	82	--	255	--
				V2	8.5			85	35E	16.0 *	-11.3	13.5	-12.8	18.5 *	-10.1	0.4	-12.0	0.4	-20.4	1.5	-58.1	82	0.0	248	-2.7
				V3	6.4			83	35C	17.2 *	-4.8	13.8	-10.4	20.1 *	-2.6	0.4	-4.9	0.6	26.5	2.6	-27.0	82	0.0	254	-0.4
22	Male	60	18	V1	8.6	162	65	85	35C	18.0 *	--	15.6	--	20.7 *	--	0.3	--	0.6	--	4.5	--	69	--	257	--
				V2	7.9			85	35C	17.0 *	-5.5	13.9	-11.1	19.3 *	-7.0	0.1	-60.0	0.5	-25.8	4.0	-12.5	71	2.9	258	0.4
				V3	7.2			90	20	18.6 *	3.4	14.0	-10.4	21.4	3.5	0.3	1.6	0.6	0.5	2.8	-39.0	70	1.4	259	0.8
23	Male	54	8	V1	7.4	131	71	90	35C	21.6	--	19.8	--	23.7	--	0.1	--	0.7	--	15.1	--	77	--	320	--
				V2	7.6			80	35C	18.8 *	-12.6	18.1	-8.9	21.3	-10.1	0.1	-0.9	0.6	-7.9	13.3	-11.8	77	0.0	324	1.3
				V3	7.6			85	35C	19.2 *	-11.0	18.2	-8.3	21.6	-8.9	0.1	-5.8	0.7	4.2	12.2	-19.1	78	1.3	325	1.6
24	Male	66	21	V1	7.7	147	73	85	35C	20.0	--	14.7	--	21.7	--	0.1	--	0.6	--	9.0	--	76	--	286	--
				V2	7.7			65	35C	19.0 *	-4.9	14.0	-4.6	20.4 *	-6.0	0.1	-1.8	0.6	-3.2	8.6	-4.6	76	0.0	287	0.3
				V3	7.3			75	43A	17.5 *	-12.7	11.6 *	-21.1	19.2 *	-11.7	0.2	99.4	0.5	-20.9	10.9	20.9	76	0.0	287	0.3
25	Male	59	24	V1	6.6	142	83	85	35C	19.5 *	--	15.9	--	21.5	--	0.3	--	0.7	--	9.5	--	82	--	262	--
				V2	6.7			90	35C	19.7 *	1.4	14.5	-8.7	21.5	0.0	0.3	-5.2	0.7	4.3	13.0	37.9	82	0.0	262	0.0
				V3	7.2			85	35D	18.0 *	-7.3	13.8	-13.5	20.5 *	-4.5	0.3	-4.6	0.7	5.1	3.7	-60.6	81	-1.2	264	0.8
26	Male	57	19	V1	7.3	125	75	85	35C	19.2 *	--	16.1	--	21.7	--	0.3	--	0.6	--	5.2	--	72	--	235	--
				V2	7.3			80	35C	19.3 *	0.2	16.8	4.8	22.0	1.3	0.3	-2.0	0.6	-2.1	3.9	-25.6	73	1.4	241	2.6
				V3	8.7			85	35C	16.9 *	-12.2	12.3 *	-23.1	19.4 *	-10.7	0.3	-5.7	0.6	-8.6	4.3	-17.1	72	0.0	236	0.4
27	Male	63	15	V1	5.3	161	83	85	35C	22.3	--	18.7	--	24.0	--	0.1	--	0.6	--	6.2	--	80	--	262	--
				V2	5.3			87	35C	22.3	0.0	16.9	-9.8	23.7	-1.4	0.1	5.5	0.6	-3.9	7.2	15.4	79	-1.3	269	2.7
				V3	5.3			85	35D	22.0	-1.3	17.4	-7.2	23.5	-2.2	0.1	7.9	0.6	-11.0	7.2	15.2	79	-1.3	260	-0.8
28	Male	67	20	V1	6.6	138	79	85	35C	19.5 *	--	15.6	--	21.7	--	0.3	--	0.7	--	2.3	--	83	--	253	--
				V2	6.6			85	35C	19.5 *	-0.1	15.9	2.0	21.3	-1.5	0.3	-5.6	0.7	-7.4	6.7	189.4	84	1.2	257	1.6
				V3	8			85	35C	18.7 *	-3.9	15.0	-3.6	20.7 *	-4.2	0.3	-14.0	0.5	-28.1	4.2	81.8	86	3.6	257	1.6
29	Male	66	24	V1	7.1	140	60	85	35C	19.8	--	17.3	--	21.8	--	0.1	--	0.6	--	11.4	--	83	--	315	--
				V2	7.1			83	35C	18.3 *	-7.5	19.0	9.9	20.2 *	-7.4	0.1	-4.0	0.6	5.1	7.4	-35.0	83	0.0	317	0.6
				V3	7.6			85	35C	17.3 *	-12.6	15.4	-11.2	19.5 *	-10.8	0.1	-2.6	0.7	11.1	5.6	-50.8	82	-1.2	316	0.3

ID	Gender	Age [yrs]	Diab. Dur. [yrs]	Visit	HbA1c [%]	Blood Pressure [mmHg]		BVCA	ETDRS	VD SRP		VD DRP		VD FR		FAZ Area		FAZ Circ		RNFL Thickness		GCL-IPL Thickness		Full Retina Thickness	
						S.	D.			[mm^{-1}]	%	[mm^{-1}]	%	[mm^{-1}]	%	[mm^{2}]	%	[au]	%	[µm]	%	[µm]	%	[µm]	%
				V1	6.7			80	35C	20.3	--	16.4	--	21.7	--	0.2	--	0.7	--	6.4	--	76	--	276	--
30	Female	63	9	V2	7.4	145	86	80	35C	21.3	5.3	17.6	7.3	22.9	5.4	0.1	-26.9	0.7	3.6	6.6	3.1	76	0.0	273	-1.1
				V3	7			80	35C	20.4	0.7	15.8	-3.9	21.9	1.1	0.1	-19.0	0.7	4.3	7.9	23.4	77	1.3	276	0.0
				V1	7.5			85	35D	18.7 *	--	13.9	--	20.4 *	--	0.4	--	0.7	--	2.3	--	76	--	233	--
31	Female	68	11	V2	7.5	146	72	78	35C	19.1 *	1.9	13.2	-5.2	20.7 *	1.5	0.4	-7.5	0.7	4.0	5.4	132.5	76	0.0	228	-2.1
				V3	7.5			80	35E	18.0 *	-3.8	11.4 *	-17.9	19.7 *	-3.5	0.4	-4.7	0.6	-1.7	3.5	51.4	76	0.0	229	-1.7
				V1	6.9			85	35C	19.8	--	14.6	--	21.4	--	0.1	--	0.6	--	8.5	--	69	--	288	--
32	Male	67	16	V2	6.9	160	70	83	35C	18.9 *	-4.8	12.7	-12.9	20.6 *	-3.6	0.1	1.1	0.5	-7.1	8.3	-2.6	70	1.4	298	3.5
				V3	6.9			85	35E	17.0 *	-14.0	10.8 *	-26.1	18.5 *	-13.4	0.1	1.8	0.5	-18.4	8.6	0.7	70	1.4	298	3.5
				V1	6.7			75	35C	16.1 *	--	10.5 *	--	18.2 *	--	0.2	--	0.4	--	6.0	--	58	--	319	--
33	Male	73	18	V2	6.7	110	54	80	35C	15.5 *	-4.2	10.6 *	1.4	17.7 *	-3.0	E	E	E	E	10.1	69.3	62	6.9	290	-9.1
				V3	7.3			76	35D	14.8 *	-8.4	10.8 *	2.7	17.1 *	-5.9	0.1	-64.4	0.5	29.5	8.6	44.6	61	5.2	304	-4.7
				V1	6.5			85	35C	21.4	--	16.5	--	23.1	--	0.2	--	0.7	--	6.0	--	90	--	249	--
34	Male	62	10	V2	8.1	146	73	85	20	21.4	0.1	16.4	-0.7	23.3	1.0	0.2	0.5	0.7	-0.3	2.9	-52.6	90	0.0	250	0.4
				V3	7.5			85	35C	19.4 *	-9.3	15.5	-6.1	20.8 *	-10.0	0.2	3.2	0.7	-8.8	3.7	-38.9	91	1.1	251	0.8
				V1	6.2			85	35D	17.6 *	--	13.7	--	19.7 *	--	0.2	--	0.6	--	9.0	--	79	--	260	--
35	Male	78	16	V2	6	133	72	82	20	17.9 *	1.7	13.1	-4.6	19.7 *	0.1	E	E	E	E	6.4	-28.9	79	0.0	264	1.5
				V3	5.9			80	35C	18.2 *	3.3	12.4 *	-9.2	19.7 *	0.1	0.3	19.6	0.6	-5.7	6.7	-26.1	79	0.0	262	0.8

ID	Gender	Age [yrs]	Diab. Dur. [yrs]	Visit	HbA1c [%]	Blood Pressure [mmHg]		BVCA	ETDRS	VD SRP		VD DRP		VD FR		FAZ Area		FAZ Circ		RNFL Thickness		GCL-IPL Thickness		Full Retina Thickness	
						S.	D.			[mm^{-1}]	%	[mm^{-1}]	%	[mm^{-1}]	%	[mm^2]	%	[au]	%	[µm]	%	[µm]	%	[µm]	%
				V1	6.6			75	43A	15.8 *	--	9.1 *	--	17.6 *	--	0.3	--	0.4	--	9.9	--	69	--	223	--
36	Female	80	24	V2	7	119	60	75	35C	15.5 *	-1.9	8.9 *	-2.4	17.1 *	-2.6	0.3	26.9	0.6	53.6	4.6	-53.9	68	-1.4	222	-0.4
				V3	7.4			80	35C	14.7 *	-6.9	8.6 *	-5.7	16.5 *	-6.2	0.3	0.5	0.3	-26.3	2.4	-76.0	68	-1.4	222	-0.4
				V1	11			80	43A	19.9	--	16.6	--	21.5	--	0.1	--	0.7	--	7.4	--	75	--	263	--
37	Male	67	21	V2	9.7	150	85	85	43A	19.4 *	-2.6	16.0	-3.4	21.3	-0.5	0.1	3.9	0.6	-9.1	7.6	2.9	76	1.3	265	0.8
				V3	12			85	43A	16.9 *	-15.0	13.8	-16.9	18.9 *	-12.1	0.1	1.1	0.6	-17.3	8.0	8.0	75	0.0	261	-0.8
				V1	7.1			85	43B	20.9	--	14.9	--	22.4	--	0.2	--	0.6	--	5.1	--	88	--	261	--
38	Male	56	17	V2	7.1	137	77	80	35E	21.1	0.6	16.2	8.6	22.6	0.9	0.2	0.5	0.7	9.2	4.1	-19.8	88	0.0	257	-1.5
				V3	7.1			85	35C	20.5	-1.8	13.2	-11.3	22.1	-1.2	0.2	-0.2	0.6	2.0	4.0	-22.0	86	-2.3	258	-1.1
				V1	7.6			80	43A	18.9 *	--	16.1	--	21.1 *	--	0.2	--	0.6	--	6.9	--	78	--	271	--
39	Male	67	16	V2	7.4	150	65	76	43A	18.3 *	-2.8	14.1	-12.5	20.9 *	-1.2	0.2	-2.3	0.6	0.6	7.8	12.8	79	1.3	267	-1.5
				V3	7.4			75	43A	14.1 *	-25.3	11.2 *	-30.6	16.5 *	-21.8	E	E	E	E	4.8	-30.6	80	2.6	277	2.2
				V1	6.6			85	43A	18.7 *	--	15.3	--	21.0 *	--	0.2	--	0.6	--	4.7	--	81	--	304	--
40	Male	59	10	V2	6.6	140	77	85	47A	19.3 *	3.4	15.9	4.1	21.1 *	0.4	0.2	-0.2	0.6	-3.2	10.7	130.8	81	0.0	313	3.0
				V3	6.8			88	43A	17.8 *	-4.9	13.5	-11.8	19.4 *	-7.5	0.1	-14.5	0.4	-36.7	5.8	25.4	80	-1.2	307	1.0
				V1	7			85	43A	21.1	--	17.9	--	22.4	--	0.2	--	0.6	--	4.6	--	97	--	294	--
41	Male	54	7	V2	5.9	139	85	85	35C	19.4 *	-8.0	14.9	-16.9	21.1 *	-6.0	0.2	13.2	0.5	-8.2	5.3	16.4	96	-1.0	295	0.3
				V3	6.2			90	35C	19.1 *	-9.5	16.3	-9.0	20.2 *	-9.8	0.2	4.8	0.5	-8.1	8.0	75.7	96	-1.0	297	1.0
				V1	7.8			80	43A	18.6 *	--	11.2 *	--	20.1 *	--	0.3	--	0.7	--	7.8	--	75	--	231	--
42	Female	67	20	V2	8.8	150	70	75	43A	17.7 *	-4.8	9.6 *	-14.7	19.4 *	-3.6	0.3	-0.4	0.7	1.3	7.1	-8.6	76	1.3	232	0.4
				V3	8.8			75	47A	17.5 *	-6.2	11.2 *	0.3	19.1 *	-5.0	0.3	-5.8	0.7	-2.4	5.8	-25.5	76	1.3	234	1.3
				V1	8.2			90	47A	19.3 *	--	14.5	--	20.8 *	--	0.3	--	0.7	--	4.9	--	83	--	259	--
43	Male	57	15	V2	8.2	146	84	90	47A	19.5 *	1.0	14.9	3.0	21.2	1.6	0.3	5.6	0.6	-15.6	10.1	105.0	84	1.2	271	4.6
				V3	7.8			90	47A	20.1	4.0	16.6	14.8	21.3	2.3	0.3	1.0	0.6	-13.1	17.6	258.8	88	6.0	274	5.8
				V1	7.4			85	43B	21.9	--	17.8	--	23.3	--	0.4	--	0.6	--	5.4	--	86	--	240	--
44	Female	55	15	V2	7	136	77	85	43B	20.1	-8.2	16.4	-7.9	21.8	-6.2	0.4	1.4	0.6	5.5	4.6	-15.2	87	1.2	242	0.8
				V3	7.8			85	43B	20.2	-8.0	15.2	-14.3	21.6	-7.1	0.4	4.0	0.6	15.2	3.5	-35.0	86	0.0	240	0.0
